# Editorial for the Special Issue on Small-Scale Deformation using Advanced Nanoindentation Techniques

**DOI:** 10.3390/mi10040269

**Published:** 2019-04-22

**Authors:** Ting Tsui, Alex A. Volinsky

**Affiliations:** 1 Department of Chemical Engineering, University of Waterloo, Waterloo, ON N2L 3G1, Canada; 2 Department of Mechanical Engineering, University of South Florida, 4202 E Fowler Ave. ENB 118 Tampa, FL 33620, USA

Nanoindentation techniques have been used to reliably characterize mechanical properties at small scales for the past 30 years. Recent developments of these depth-sensing instruments have led to breakthroughs in fracture mechanics, time-dependent deformations, size-dependent plasticity, and viscoelastic behavior of biological materials. This special issue contains 11 papers covering a diverse field of materials deformation behavior. Müller et al. [1] developed a new nanoindentation method to evaluate the influence of hydrogen on the plastic deformation of nickel. Effects of radiation on ferritic-martensitic steels were studied by Roldán et al. [2]. The applications of the depth-sensing indentation method in the mechanical reliability of microelectronic packaging products, such as through-silicon via (TSV) structures and lead-free solder, were performed by Wu et al. [3] and Long et al. [4], respectively. Gan et al. [5] and Chiu et al. [6] investigated the fracture behavior of cementitious cantilever beam and InP single crystals. Studies of nanometer scale deformation of metallic glass materials (Zr-Cu-Ni-Al and La-Co-Al alloys) [7] and Bi_2_Se_3_ thin films [8] were also part of the collected manuscripts. The mechanical deformation of mammalian cells and other biological materials [9,10] were also discussed in this focus issue. Influence of surface pit on the nanoindentation was studied by Zhang et al. [11]. The editors would like to thank these authors for their contributions to this focus issue.
